# Clinical and Molecular Characterization of ALG1-CDG

**DOI:** 10.15844/pedneurbriefs-30-2-5

**Published:** 2016-02

**Authors:** Radhika Dhamija, Chelsea Chambers

**Affiliations:** 1 Department of Neurology (Division of Pediatric Neurology), University of Virginia, Charlottesville, VA

**Keywords:** Asparagine-Linked Glycosylation Protein 1, CDG, Carbohydrate-Deficient Transferrin, Xeno-Tetrasaccharide

## Abstract

Investigators from the Human Genetics Program, Sanford Burnham Prebys Medical Discovery Institute, La Jolla, California and a large study group utilized a combination of exome sequencing, targeted gene panels, and Sanger sequencing to identify thirty-one pathogenic variants in thirty-nine affected individuals with ALG1-CDG from 32 families.

Investigators from the Human Genetics Program, Sanford Burnham Prebys Medical Discovery Institute, La Jolla, California and a large study group utilized a combination of exome sequencing, targeted gene panels, and Sanger sequencing to identify thirty-one pathogenic variants in thirty-nine affected individuals with ALG1-CDG from 32 families. Congenital disorders of glycosylation (CDGs) cause impaired synthesis of glycoconjugates and are genetically heterogeneous with pathogenic variants in over one hundred genes that can cause it. ALG1 mutations cause a rare autosomal recessive disorder called ALG1-CDG. Of the thirty-one pathogenic variants reported, twenty-two (65%) are novel variants. This study has tripled the number of known patients with ALG1-CDG.

Pathogenicity of each mutation was confirmed based on its inability to rescue impaired growth or hypoglycosylation of a standard biomarker in an alg1-deficient yeast strain. ALG1-CDG patients are known to accumulate a novel N-linked xeno-tetrasaccharide. Since this tetrasaccharide does not normally occur in mammals and is primarily detected in ALG1-CDG cases, it serves as a biomarker for either detecting or confirming a diagnosis of ALG1-CDG. This biomarker was identified in all 27 patients tested.

This study also expands the clinical phenotype of ALG1-CDG patients. All ALG1-CDG patients have substantial neurological involvement; however other systems are variably affected. Several factors may cause this variability, including how ALG1 mutations affect residual mannosyltransferase activity or GDP-Man binding efficiency, complex formations with other proteins, tissue specific expression, or even protein stability. [1]

COMMENTARY. Congenital disorders of glycosylation are a group of predominantly autosomal recessive diseases with multi-system affects ranging from developmental delay and hypotonia with multiple organ system involvement to hypoglycemia and enteropathy [1]. Congenital disorders of N-linked glycosylation are of two types. Type I involve defects in either the synthesis or transfer of a dolichol lipid –linked oligosaccharide to proteins in the endoplasmic reticulum while type II result from golgi dependent processing of protein bound oligosaccharide. The most common type of CDG is PMM2-CDG, while the other types are rarer. The screening test for almost all types of CDG-N-linked is transferrin isoform analysis; however molecular testing is needed for confirmation [2, 3]. Next generation sequencing technology (NGS) has enabled novel disease gene discoveries and was used in this study to identify cases with this are disorder (ALG1-CDG).

The clinical features in this group of 39 patients included developmental delay, hypotonia, epilepsy, microcephaly, cerebral and cerebellar atrophy, strabismus, dysmorphic facial features, gastrointestinal abnormalities and hypoalbuminemia [1]. Thus a diagnosis of CDG's should be considered in a child presenting with developmental delay and multi-system organ involvement. For genetically and phenotypically heterogeneous disorders like CDG's, targeted panel based sequencing should be done if the screening transferrin isoform analysis is abnormal. If negative, it can be followed by exome sequencing to increase the diagnostic yield. This principle has helped find a genetic basis in a variety of undiagnosed neurological disorders in a cost effective manner. This approach also helps overcome some unique diagnostic challenges in CDG's such as: genetic heterogeneity (large number of causative genes) and phenotypic heterogeneity (multiple genes with overlapping) [1–3].
